# Contingency and paradoxes in management practices—development plan as a case

**DOI:** 10.1108/JHOM-06-2022-0175

**Published:** 2024-02-29

**Authors:** Erlend Vik, Lisa Hansson

**Affiliations:** Faculty of Business Administration and Social Sciences, Molde University College, Molde, Norway; Faculty of Logistics, Molde University College, Molde, Norway

**Keywords:** Development plan, Strategic planning, Joint planning, Specialist care, Management, Norway, System theory

## Abstract

**Purpose:**

As part of a national plan to govern professional and organizational development in Norwegian specialist healthcare, the country’s hospital clinics are tasked with constructing development plans. Using the development plan as a case, the paper analyzes how managers navigate and legitimize the planning process among central actors and deals with the contingency of decisions in such strategy work.

**Design/methodology/approach:**

This study applies a qualitative research design using a case study method. The material consists of public documents, observations and single interviews, covering the process of constructing a development plan at the clinical level.

**Findings:**

The findings suggest that the development plan was shaped through a multilevel translation process consisting of different contending rationalities. At the clinical level, the management had difficulties in legitimizing the process. The underlying tension between top-down and bottom-up steering challenged involvement and made it difficult to manage the contingency of decisions.

**Practical implications:**

The findings are relevant to public sector managers working on strategy documents and policymakers identifying challenges that might hinder the fulfillment of political intentions.

**Originality/value:**

This paper draws on a case from Norway; however, the findings are of general interest. The study contributes to the academic discussion on how to consider both the health authorities’ perspective and the organizational perspective to understand the manager’s role in handling the contingency of decisions and managing paradoxes in the decision-making process.

## Introduction

Over the last few decades, all Nordic countries have engaged in hospital reforms (Kirchhoff *et al*., 2019). Shifting the focus from new public management to new public governance, various management practices and principles have been introduced to restructure healthcare (Pollitt and Bouckaert, 2004; Peters and Pierre, 2004; Osborne, 2006), affecting core aspects of healthcare organizations. A central tension in these management practices is the autonomy and control of such organizations; this includes their management, identities, roles, performance, accountability and coordination (Lægreid *et al*., 2008).

In this article, we focus on one management practice—*development plans*. As part of a national plan to govern professional and organizational development in Norwegian specialist healthcare, all specialist healthcare organizations were tasked with making their own development plans. The goal behind this requirement is to create a common vision and a strategy for the future and foresee and implement measures that can meet future challenges (St.meld.11, 2015-2016). More specifically development plans should describe the organization’s current situation, its challenges and future goals. And most importantly, how these goals could be reached through different measures and prioritizations. Similar management approaches are seen in several other public sector organizations in the Nordic countries. The rationale is that the organization itself should be part in formulating the goals by which it will be measured. This is especially current in healthcare organizations. Healthcare organizations hold a profession-led organizational culture, and the professional competence legitimizes the staff’s ability to make and implement decisions (Törner *et al*., 2020).

A development plan involves elements of strategic planning (Bryson, 2004, p. 6), aiming to describe “what an organization is, what it does, and why it does it.” It is influenced by New Public Governance, with elements of joint planning (Nicholson *et al.*, 2013; Osborn, 2006). The goal is to integrate different levels of specialist healthcare and primary healthcare by ensuring that employees and other stakeholders work toward common goals. Development plans are interesting in light of current public sector reforms because they highlight the tension between control and autonomy (Lægreid *et al*., 2008). On the one hand, governments use them to control organizational and professional development. On the other hand, the development plan represents an organization’s autonomy—its self-defined purpose—and is supposed to be developed within the organization.

This paper argues that one should consider the perspective of health authorities and adopt an organizational perspective when studying management practices. Using the development plan as a case, it analyzes how managers at the organization level navigate and legitimize the planning process within their own organization and externally with a range of stakeholders. Such a perspective highlights the institutional complexity and the presence of multiple logics in the plan process (Høiland and Klemsdal, 2020). The navigation and legitimization by managers correspond to a central issue recognized in organizational theory: how organizations handle the contingency of decisions and paradoxes in decision-making processes (Knudsen, 2006; Jansson *et al*., 2021).

To capture the institutional complexity and different rationalities within a healthcare system, specifically those rationalities evident in the development plan process, the paper combines Luhmann’s systems perspective with management theory. Luhmann’s (2012) thesis on functional differentiation is used to capture the complexity of the context surrounding the work process and the challenges of involving and integrating various actors. Management theories, specifically theories of inclusive management, permit an analysis on how the manager in a functionally differentiated healthcare system (Vik and Hjelseth, 2022) tries to facilitate participation and inclusion in the work process of constructing a development plan. Which by various levels of the government is seen as important part of the plan process and crucial for reaching the goal “a common vision and a strategy for the future” (St.meld.11, 2015-2016).

The aim is divided into the following research questions:

RQ1. How do local (clinical-level) managers organize, navigate and legitimize the process of making a development plan?

RQ2. How can we further understand the role of managers in dealing with contingency of decisions when applied to organizational work with development plans?

The paper is organized into five parts. The theoretical section introduces Luhmann (1993) theory on social system and discusses ways to integrate inclusive management theories to complement Luhmann theory. The methodological part describes the single case study method and the variety of materials used to capture the complexity of the case. The results follow the clinics process of working with the development plan. In this section, theoretical concepts are used to further explain the events. The paper ends with a discussion and a section that points out the main conclusions drawn from this research. 

## Theory

### A functionally differentiated healthcare system

Functional differentiation is central in Luhmann’s (2012) system theory. Functional differentiation is the division of modern society into multiple functionally specialized and autonomous subsystems, all managing specific functions for society. In this paper, we argue that functional differentiation is the primary form of differentiation in the healthcare system. Describing the healthcare service as functionally differentiated implies that it is one system comprising several autonomous and self-referencing subsystems, each maintaining its function for the healthcare service as a whole. “Subsystems” refers to the various professions, organizational units and administrative levels in the overall healthcare system. Such a perspective places a stronger focus on the autonomy of subsystems than on formal and hierarchic organizational structures in the healthcare system (Vik and Hjelseth, 2022). The central subsystem for this paper is an organization unit, a clinic for mental health and substance abuse in the Norwegian specialist health service.

The cornerstone of Luhmann’s (1993) systems theory is its distinction between a system and an environment. Furthermore, Luhmann describes a system as being operatively closed, implying that any operation is always the result of conditions of possibility determined within the system itself. Any action or decision taken by the system is based on the system’s own logic and understanding. This insight implies that at all development plans are created inside the relevant subsystems. In its most basic form, the development plan can be understood as a way of operationalizing an organization’s function: defining its purpose, addressing the reasons for its existence and articulating what it wants to achieve (Bart and Tabone, 1998). Hence, using Luhmann’s (1993) concept, the organization can use the development plan to define itself in relation to its environment.

Functional differentiation also points to the structural coupling between the healthcare service and the various functional systems in society. A functional system is an abstract communication system that an organization attaches itself to by making decisions. Each functional system refers to its own logic, rationality and communicative structures (Luhmann, 2012). Economic, political and health systems are examples of functional systems that employ different criteria for observing the world. Table 1 shows the various codes, mediums, programs and functions that organizations can activate through functional systems.

The basic premise of Luhmann’s (2012) work is that the differentiation process of modern society entails the crystallization of organizations that are attached primarily to one functional system. Political parties and public administration communicate through the political code while banks and businesses communicate through the economic code. Many modern organizations attach themselves not to one primary functional system but to multiple functional systems. This phenomenon is especially evident in public sector organizations, where the demands of the different functional logics clash—sometimes violently, sometimes nearly invisibly, sometimes harmoniously, but always, inevitably, as differences (Knudsen and Vogt, 2014). This “polyphony” means that organizations of the same type could, in principle, attach themselves to different functional systems with crucial effects on their communicative structures. The functional system to which an organization chooses to attach itself when making a decision will have consequences for how it communicates and for how the organization fundamentally evolves (Åkerstrøm, 2002). In the present case, it makes a difference whether healthcare organizations attach themselves to the health, economic, or political system when communicating about their development plans. Different functional systems cannot understand one another’s rationalities and evaluation criteria, and this can be a central source of tension in the process of constructing a development plan. In the process of constructing a development plan a healthcare organization must address the needs of stakeholders representing different functional systems. This requirement is central in light of systems theory because an organizational system creates itself by forming an internal structure that mirrors its environment (Luhmann, 1978). An organization’s self-description thus greatly depends on how it constructs its environment. In constructing an image of its surroundings, the organization concomitantly constructs an image of itself. In the present case, a healthcare organization is not only one system within one environment but often operates within several systems and environmental constructions. Accordingly, one must observe how such organizations communicate multiple systemic environmental demarcations in their development plan work.

### Merging Luhmann’s organization theory with management theory

This study observes how managers handle the tension between communicating demarcations from multiple systems and environments and the tension between control and autonomy on an organizational level, using development plan work as a case. In systems theory, organizations are seen as social systems that can stabilize forms of action and behavior by *deciding* about stronger or weaker conditions for practices and procedures (Luhmann, 1978). In other words, organizational systems operate through decisions and decision communication.

A key theme in Luhmann’s (1978) organization theory concerns how organizations manage the contingency of decisions. The contingency of decisions points to the fact that a decision is neither necessary nor certain but could always have been made differently. This contingency makes connectivity less likely because it calls into question the notion of connecting to a decision that could inherently have been made differently (Knudsen, 2012). Connectivity is essential to decisions because it is only the connection to further decisions that can turn a decision into a real decision. A decision to which no further decisions are connected turns out not to be a decision at all, but just noise.

Knudsen (2012) points out that a main strategy for managing the contingency of decisions in organizations is *displacement*. For example, an organization can “deparadoxify” its decisions by interpreting them to external stakeholders as necessary responses, displacing the contingency onto the environment. Another way is to displace the contingency onto decision-makers, such as the managers of the organization. Securing legitimacy within the organization regarding how decisions related to the development plan are deparadoxed will be crucial for the connectivity of future decisions.

To observe how organizations handle the paradox of decisions, this paper uses the concept of inclusive managers and brokers. Inclusive management brings together participants from different practices in collaborative settings. Such a collaborative setting could be important in the process of creating a development plan since the statement must be anchored in the organization’s employees to be legitimate and successful (Klemm *et al*., 1991).

A manager can assume the role of promoting, as well as inhibiting, inclusion. An inclusive manager tries to design inclusive processes and create a community of participation in which people can share information and perspectives and work together. However, it is not enough simply to bring people together; the people must also be willing to listen and be engaged in ways that advance the collaborative process, in which inclusive managers play a key role. Inclusive managers identify various relevant areas and know the problems faced in each one. They encourage people to see different perspectives in discussions or meetings, fostering an atmosphere in which problem-solving occurs. However, these meetings do not necessarily enable people to feel connected or to trust one another, so joint activities in this sense can be either constructive or destructive. Managers must try to create joint activities that provide a shared experience and transcend boundaries between participants (Feldman and Khademian, 2007).

Kimble *et al*. (2010) demonstrate that inclusive processes do not have to be driven by managers and emphasize the role of “brokers.” The work of brokers is similar to that of inclusive managers, as brokers make coordination possible by opening up new possibilities for learning and exchange. Brokers help other actors transfer, translate, or transform the meanings encountered during joint activities (Carlile, 2004). A development plan in a functionally differentiated healthcare system must address various perspectives and the needs of different external and internal actors. Translating and transforming meanings and knowledge between these actors is essential in the process of making and legitimizing the plan.

A broker translates knowledge created in one group into the language of another so that the new group can integrate it into its cognitive portfolio. To do this, brokers must be able to manage the relationships between individuals and act as translators. The broker’s role necessitates a delicate balancing act. To be effective, brokers must have authority in all groups to which they belong. They must be able to evaluate the knowledge produced by the different groups and earn the trust and respect of the various parties involved. Over time, the broker’s activities may lead to the development of a repertoire of shared resources, such as the rules and procedures used by the group (Kimble *et al*., 2010).

The presented theory will be used to identify and show the complexity of development plan work in a functionally differentiated healthcare system and highlight that the plan process opens up for tensions between different logics. The concepts of inclusive management and brokers will be used to analyze how organizations handle this complexity and how managers navigate and legitimize decisions related to the development plan within the organization.

## Method

The research design is based on a single case study of a clinic’s work with a development plan. The material is rich. To identify the intentions of the plan from a government perspective, a larger content analysis of central documents related to development plan work in the context of Norwegian specialist healthcare was conducted. The national government’s intentions for the development plans were analyzed, as was the way in which these intentions were translated to the organizational (clinic) level. As a second step, an in-depth analysis (case study) of development plan work at the clinical level was conducted. The selected case is that of a clinic dealing with mental health issues and drug addiction. This clinic was “under construction,” meaning that it had recently been reorganized with a new structure, as two clinics (a mental health clinic and a drug addiction clinic) had been merged into one. This organization is segregated into five units and 27 sections with approximately 1,000 employees. The clinic serves a large geographical area, and there had been economic and professional tension between the geographically separate organizational units. This new clinic was, therefore, seeking to define its culture, work processes, vision and so on. Due to the re organization, we argue that the case can be seen as an “extreme case” (Flyvbjerg, 2006, p. 23). An extreme case is relevant for this study because such a case involves the possibility of constructing a development plan based on a “blank page.” A more established organization might have used a “copy and paste” strategy, reproducing older strategy documents and plans.

**Figure 1:**
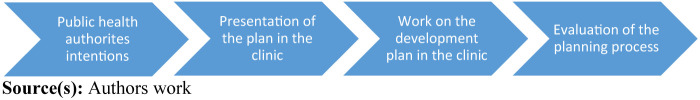
Timeline of case events

The research investigating the work with the development plan ended when the organization had constructed its plan. After finalization at the clinical level, the development plans are sent to the upper organizational level; these plans are then used as a foundation for the local and regional development plans (see nos. 7–9, Figure 1). However, this part of the process is not covered in the present article; our focus is on the organizational unit of the clinic.

At the clinical level, group observations, individual semi-structured interviews and public documents were used as data sources. Observations were made at three strategic management meetings in which the development plan was discussed. Group observation made it possible to capture ongoing discussions within the development plan work, especially problems with the work and content negotiations and the managers’ various roles and influences in the process. The meetings were held 2–3 months apart and represented different parts of the plan process.

Ten key people involved in the work process were selected for in-depth individual interviews: the clinic’s adviser (who was given internal responsibility for development plan work), four unit managers and five section managers. Interviews with the unit and section managers were conducted during the process of developing the plan. An interview with the clinic’s adviser was conducted after the evaluation of the work process. The interviews were conducted in Norwegian and were audiorecorded and transcribed. The project was ethically evaluated and approved by the Norwegian Centre for Research Data.

This research employs a qualitative research design, which combines different data gathering methods (interviews, document research and observation). Qualitative data triangulation has several purposes. The combined data sources contribute to a “thick” and complex description of the studied case or phenomenon. Data triangulation is also an important method of ensuring validity (Bryman, 2016). In our research, interviews were used to further explore discussions that were observed or to investigate how statements in public documents are operationalized by different actors.

O'Reilly and Kiyimba (2015, p. 96) discuss the challenges of combining qualitative approaches and distinguish between “mixed qualitative methods” and “synthesizing methodologies.” Our approach uses a mixed qualitative method, which is a single qualitative study operating within a singular methodology but using more than one method of data collection. This approach means that data from different methods are analyzed through the same analytic framework and are thus epistemologically congruent. In our study system theory and the concept of inclusive management guided the analysis of the empirical data; for example, the emphases of the different codes and system boundaries were identified, as were the actions taken by the managers and presumptive brokers to achieve an inclusive process at the clinical level.

## Results

The following section presents the work with the development plan at a clinic for mental health and drug addiction. Through the section, theoretical core concepts will be applied to bring forward explanations of events that occur in the case study. The section is divided into four parts representing a timeline of different stages in the plan process (see Figure 1).

(1) Public health authorities guidelines regarding the development plan and how these intentions are translated at different levels (2) The clinic’s reactions to the requirements of constructing a development plan (3) how the clinic organized the plan work; and (4) evaluated the planning process.

### The multilevel context of work with development plans

**Figure 2:**
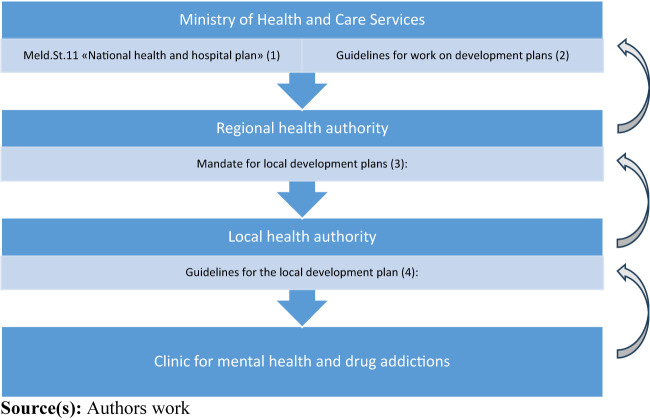
A multilevel plan process

As part of implementing the National Health and Hospital Plan, all health authorities have had to create their own *development plans.* This means that a standard approach is set by the government and then adapted on various government levels down to the clinic level. This section examines how government intentions are translated and operationalized through various government guidelines within Norwegian specialist healthcare down to the clinic level. Figure 2 illustrates the multilevel plan process and the different documents that were produced to secure connectivity throughout the different levels of the healthcare authorities. The overall guidelines on how to manage development plans were constructed by the Ministry of Health and Care Services and distributed to the five regional health authorities. The regional health authorities then translated the national guidelines according to their mandate, serving as a premise for the work on local development plans. Based on their mandates, the local health authorities created their own guidelines. After finalization at the clinical level, the development plans are sent to the upper organizational level; these plans are then used as a foundation for the local and regional development plans. These plans then make the foundation for the national development plan. This process is not covered in this article.

Luhmann’s (1993) theoretical concepts of connectivity and contingency are used to analyze decision communication regarding development plans in what we call a multilevel translation process. The Ministry of Health Care Service clearly states the political goals behind the development plan. The political goal of the guidelines is to achieve collectively binding decisions through the development plan formulated by the various authorities, clinics and hospitals. The guidelines present a recommended thematic structure for the development plans, whose main components are to be historical background, current situation, contextual change, desired situation and strategic choices. The guidelines also emphasize transparency and stakeholder involvement. All documentation connected to the development plan process is therefore published, and at all levels there should be broad involvement of users, patient organizations, professionals, unions, municipalities and private actors. The emphasis on involvement and transparency can be understood as a way of legitimizing the political system’s function of achieving collective, binding decisions regarding healthcare strategy. Hence, the national guidelines for development plans should help in navigating the practical work. The guidelines for the plans are then translated to the regional health authorities.

The regional health authorities stated that local development plans must follow national and regional instructions issued through various parliamentary reports and reforms. This includes guidance from the National Health and Hospital Plan (St.meld.11, 2015-2016) and the Coordination Reform (St.meld.nr.47, 2008-2009). The regional instructions concentrate on how local health services can be adapted to improve regional economic conditions, efficiency, capacity and competence. They also emphasize ways to improve internal coordination and collaboration with other health regions. The case is a clear example of how the health authorities, through the regional mandate (3), try to ensure connectivity to both the National Health and Hospital Plan and previous reforms. At the same time, there is a shift in focus from a national and political context to a regional and economic context. The goal of the development plan is no longer merely to create binding decisions regarding strategy but to create binding decisions regarding strategy based on regional economic conditions. In light of systems theory, the regional mandates entail incorporating the economic code into development plan work.

The local health authorities then created their own guidelines on how to construct perform the development plan (4). Connectivity is promoted by stating that the local development plans must follow the thematic structure recommended in the national guidelines, but we also see that the local health service authorities emphasize that the local development plan must be based in the different clinic perspectives. The focus is no longer only on general political goals and economic frames; it includes practical implications for patient treatment, the organization of different services, coordination, staff competence and technology. This brings an enormous amount of complexity into the plan process at the clinical level that can foster contingency.

### Introducing the development plan

A central actor throughout the work with the development plan at the level of the clinic is the clinic manager. In the case study, the first time the clinic manager presented the idea of making a development plan was at a leaders’ meeting at which various managers and union representatives were present. The clinic manager began by informing the participants that the clinic needed to address strategy and development. He then presented his view of the development plan, which he called “a political requirement,” the aim of which was to obtain an overview of how the various health organizations organize their work at the national, regional and local levels. The clinic manager also highlighted the thematic structure in the national guidelines (1). He specifically highlighted the clinic and the local health authority’s *economic conditions* and proclaimed that “a key question when working on the development plan is how we can get as much health for the patient as possible given the economic constraints that we work under. There have to be strategic prioritizations” (Observation 1).

The clinic manager’s presentation illustrated how the various guidelines ensured connectivity. The main translations at the different levels of the health authorities, shown in the previous section, were all covered in the presentation. By calling the development plan a *political requirement*, the clinic manager activated the political system, placing the contingency of further decisions within the context of a concrete decision premise, namely, the political guidelines. The clinic manager’s emphasis on economic conditions also implied that the economic code should be activated when working on the development plan.

During and after the presentation, there was a range of reactions from both the union representatives and various managers. There were negative reactions related to the political establishment of guidelines through a top-down process lacking professional involvement. For example, one section manager asked why politicians, instead of their own healthcare professionals, were allowed to formulate guidelines for the clinic’s development plan. Another section manager argued that “decisions on the future direction of the clinic can’t be made only at the top of the system, as the professionals working with the patients must be involved in the process.” These reactions were connected to the strong use of the political code when presenting the development plan. The way the clinic managers set the contingency of decisions within the context of the politically defined guidelines also resembled a top-down process. By emphasizing the role of healthcare professionals within the organization, the reactions sought to place decisions regarding the plan inside the organization itself, in this way activating and reinforcing the functional system of health in the ongoing process.

Based on observations of the presentation of the development plan (Observation 1), there was clearly a gap between the organization’s top management (e.g. clinic and unit managers) on the one hand and the section managers and union representatives on the other. Top management was more comfortable with political rhetoric and working to achieve political and economic goals set at the national and regional levels—or, using Luhmann’s (1993) concepts, attaching their communication to the political and economic function system. Section managers and union representatives, on the other hand, linked themselves more to the functional system of health. Discussion in the meeting addressed the fact that the organization had not yet decided how to manage the contingency of decisions connected to the development plan, nor had they chosen the functional system through which the decisions should be made.

### “Making our plan”

This section presents the “hands-on work” of the development plan, constructing the content of the plan and attempting to engage healthcare professionals. In this section, systems theory is complemented with the theoretical concepts of “broker” and inclusive management to analyze the manager’s role in facilitating participation and involvement when formulating a development plan.

Four weeks after the development plan work was first presented, another meeting was held, led by the clinic manager and a clinical adviser, who were responsible for writing and coordinating the clinic’s development plan. The clinical adviser started the meeting by stating that it was important that the development plan was based on a mutual understanding of how it could be used in the clinic. The work process must be driven from within the clinic, and the local context must be in focus. He also emphasized that the work must end up with something more than merely a political document (Observation 2). Here, the clinical adviser was alluding to inclusive management (Feldman and Khademian, 2007) and attempting to translate the development work from a political requirement into an internal, collaborative practice. To reach this goal, he had to translate and coordinate the political and economic aspects of the various guidelines into a language with which the various organizational actors could relate. In the interviews, conducted after the meeting, the clinical adviser followed up on his role and reflected on the process of making the development plan:

For me, it was very important that our plan reflect our organization and the professional work done throughout our organization. I was concerned that this was not happening. Several times I tried to make the point that it had to be *our* process. Our process of actually figuring out what our patients’ needs would be in the future and how we should meet them. I felt in the meeting, and several times afterwards, that this point wasn’t getting through. (Clinical adviser)

The clinical advisers’ reflections touch on how the strategy of displacing the contingency of development plan decisions in the organization’s environment had failed. To secure further connectivity the contingency of development plan decisions must be placed inside the organization and connected to the professional practice and not only political and economic goals.

At the meeting, the clinic manager stressed the importance of having a development plan, pointing out that the clinic lacked a clear purpose and direction and often made reactive and rash decisions instead of anticipating situations before they arose. The clinic manager argued that the development plan could be a tool for turning around this dynamic. In addition, the clinic manager wanted the development plan work to be a collaborative practice and emphasized the importance of involving the whole organization. He stated that it was a personal choice to become involved but that the managers should try to facilitate discussion about where the focus should be regarding local challenges and areas of improvement. He also pointed out that the management group (clinic manager and unit managers) could not produce a good plan by themselves (Observation 2).

Both the clinical adviser and the clinic manager emphasized the importance of translating the development plan work from the political to the local context. They also pointed out that all managers were responsible for encouraging involvement in and enthusiasm for the plan process throughout the organization. This meant that all managers (unit and section managers) in the organization were encouraged to assume the role of “brokers” (Carlile, 2004). This role was also highlighted through the method by which the clinic organized the work process of writing the development plan. To involve the whole organization, it was decided that the work process should follow the “line principle.” The goal was to use the existing organization and arenas within the organization to produce the development plan. This meant that all unit managers were to use their hierarchical lines of authority to produce a description of their current situation, possible challenges and future goals and to ensure broad involvement. In this work process, the unit managers had to involve their section managers, who would then involve their healthcare professionals through various staff and union meetings.

The management’s goal of involving the healthcare professionals in constructing the development plan was not quite achieved. The line principle did not have a positive impact on involvement, and the managers did not succeed in their job as brokers. Based on the interviews and observations, it seems that the line principle contributed to the feeling that the plan work was a top-down process. In the interviews, the section managers responsible for involving the healthcare professionals said that it was difficult to get any feedback and enthusiasm from them:

There has been little talk of the mission plan in our section. The goal of the work process was that it should be bottom-up. Yes, it was, and there has been a lot about that goal and I think many are tired of the whole thing. Especially those at the bottom. I found it hard to get any feedback or engagement. (Section manager 2)

For the development plan, everybody was supposed to be involved, and our clinic has over 1,000 employees …. There are guidelines from the Ministry of Health and Care Services about what we are to deliver and from Central Health Norway through the mandates. These quickly met with skepticism and indifference. For the employees and many of the managers, the work feels like a duty, and then people lose their commitment and enthusiasm. This has been top-down, not bottom-up at all. We are invited in, but too late, when everything has already been defined. (Section manager 3)

The ambivalence evident at the meeting was due to the fact that the development plan was part of the larger political health policy project while being an essential part of the clinic’s strategic development. In response to criticism that too much planning and too many processes were occurring at the same time, the clinic manager argued that a new process must not displace old processes. There is a political demand upon which the clinic must deliver, and it should be integrated into the clinic’s strategy work. The clinic manager noted that the work could also be used to obtain a full picture of the main challenges and collective goals to address (Observation 2).

Here the challenge of translating the political requirements into local strategy work is obvious. The clinic manager attempted to stress the importance of the development plan, but he also called it a political demand that they might as well try to use positively. The clinical adviser also noted the contradiction between a top-down demand and a bottom-up strategy process, feeling that this had made it difficult to foster involvement and commitment from the professionals:

Another element of this is that the plan is part of a bigger political order. Everybody knows that our plan will be almost invisible in the local and regional plans. Maybe we will be able to find traces of it, but its essence will disappear. This makes it more difficult to get commitment from the professionals. But it doesn’t change the fact that we need this kind of plan for ourselves. So for me, the *process* in the clinic of working on such a development plan may be more important than the document that we will send to the local health authorities. (Clinical adviser)

### Evaluation of the plan process

The last phase was to finalize the development plan. As in previous phases, to obtain broad input and legitimize the content, the finalization of the development plan was discussed at a managers’ meeting. At this meeting, all the managers and union members representing the various professions were present. The objective was to present and discuss the feedback on the development plan work process and to discuss which areas should be prioritized in the final version of the statement.

The meeting started with a presentation by the clinic manager. He stated that the development plan had been introduced as part of a governing process for professional and organizational development in clinics all over the country. This meant that decisions regarding professional and organizational change must be reflected in the development plan before they could proceed. The development plan should, therefore, be the basis for future decisions. This reflects the desire of governing bodies and healthcare authorities to use development plans to ensure connectivity in decisions concerning organizational and professional development. For the clinic manager, it was therefore important that certain key areas in the organization be prioritized. The clinic manager argued that the work that had been done so far did not constitute a good basis for assessing what professional and organizational changes were needed for the future. He stated that to make this assessment, the professionals must be more involved in the ongoing development work (Clinic Manager 2, Observation 3).

One section manager questioned the clinic manager’s thoughts on involvement when it came to decisions on prioritizing:

The national government has given us an order. We cannot prioritize 19 areas. It is a managerial responsibility to decide what is to be prioritized. We cannot have a democratic process on this …. There are so many different motives and wishes in the clinic that it must be up to the leaders to decide what our focus should be. At the same time, there are quite clear [political] guidelines about what we really should prioritize. (Section manager 2)

This discussion was concerned with how the clinic should manage the decision contingencies connected to the development plan. The section manager made the point that without displacing the paradox or handling the contingency (Knudsen, 2012), there would be no decisions: the various subsystems were not motivated to understand one another and were therefore incapable of reaching an agreement on what should be prioritized in the organization. In the interviews after the meeting, the section managers criticized the work process, calling it “skin-deep democracy”:

I think the process is being contaminated by “skin-deep democracy” when it should have been [a matter of] good strategic management. The belief in democracy and involvement is “in the time,” but not all administrative decisions should be made bottom-up. Administrative changes and organizational goals should be decided at the top. It can be unpleasant, but you cannot make good strategic decisions if you expect everybody to be involved. (Section manager 2)

The clinic manager responded:

I have to follow the assignment given to me. If I don’t, I have to find another job, but it is also important to get the process right. I can’t and won’t decide everything alone. That’s why I think it’s important to involve the whole clinic. When the time comes, everyone should have had the opportunity to get involved, and then I will make a decision. (Clinic manager 2)

At the meeting, the clinic manager opened up a discussion of how the organization should follow up on the development work. He asked whether there was any real desire to get involved and what was needed to get the healthcare professionals involved. These questions started a discussion of how the clinic should proceed to ensure the engagement of the healthcare professionals in the future process (Observation 3).

Several union representatives expressed their views on how to involve the healthcare professionals. One union representative stated that they found it difficult to get involved in the process because they could not relate to the general matter of clinic strategy. Another union representative added that there was no shortage of commitment from the professionals in regards to working with patients but that it could be difficult to get them involved in general organizational matters (Observation 3). This feedback shows that the managers did not succeed in their brokering role (Kimble *et al*., 2010). In the previous meeting, it had been stated that it was the managers’ responsibility to translate the “general” aspects addressed by the various guidelines into more concrete elements concerning the professionals’ local context and practices.

A unit manager then pointed out that the clinic has many arenas in which to facilitate broad and open processes and asked how these could be better used to achieve broader involvement in finalizing the development plan. Another union representative agreed, believing that it would be easier to involve the healthcare professionals in strategy and development questions if they had arenas in which they could discuss them, rather than simply being told by the management to get involved in something (see Observation 3). It was also pointed out that healthcare professionals cannot be seen as a homogeneous group. For example, one section manager stated that it was important to involve the professionals but did not believe that meetings between the various specialists would lead to any unity or mutual understanding because there was too much professional disagreement in the organization. One unit manager responded that it would be unfortunate to gather the different specialist groups separately, as this would only reinforce the differences between them (see Observation 3).

The point being discussed here concerns the functional differentiation in the clinic and how this challenges the goal of producing a “mutual” mission and development plan. A mission statement should define an organization’s unique and enduring purpose (Bart and Tabone, 1998). The problem for the clinic was that the various subsystems represented by the organization’s sections and professional disciplines operated according to different purposes and understandings based on their functions in the clinic. This theoretical point was exemplified in the interviews when two of the unit managers reflected on the heterogeneous group of professionals working in the clinic:

To achieve good collaboration in the clinic, we have to break down the professional boundaries between drug addiction, psychiatry, and rehabilitation and the geographical boundaries between north and south. (Unit manager)

The problem is that our focus is on ourselves and not on the clinic as a whole. Everyone looks at the clinic based on their own sections and unions. The goal must be to achieve a shared understanding … or that we should at least relate to the clinic as a whole. The goal must be to bring about a common culture with the patient in the center (Unit manager 2).

Both comments show that the mental health and drug addiction functions consist of different subsystems, both organizationally and professionally. These different subsystems operate according to different understandings and cultures, making it difficult to achieve uniform understanding and consensus when it comes to describing the organization’s challenges and long-term goals—elements that are essential for developing a strategic plan (Baetz and Bart, 1996).

## Discussion

This paper has focused on development plans as management practices as part of reform work in the healthcare sector. Development plans are interesting in light of current public sector reform changes because they challenge the tension between control and autonomy (Lægerid *et al.*, 2008). On one side, they are used by governments to control organizational and professional development. On the other side, the development plan represents an organization’s autonomy—its own defined purpose—and the plan is supposed to be developed within the organization. Using the development plan as a case, this paper has been driven by two research questions: RQ1. How do local (clinical level) managers organize, navigate and legitimize a development plan process (in a functionally differentiated system)? and RQ2. How can we further understand the role of managers in dealing with contingency of decisions when applied to organizational work with development plans?

The results show that a clinic’s development plan work is part of a broader context and that the intentions of the national and regional authorities influence the work at the clinical level. The emphasis placed by the clinic manager and the clinical adviser on involving the whole organization in the plan process is an example of inclusive management (Feldman and Khademian, 2007). Despite this inclusive approach, the managers did not succeed in involving the healthcare professionals in the development plan process.

The study identifies two reasons for the difficulty with inclusion: (1) challenges in managing the *contingency of decisions* and (2) the tension between autonomy and control. Knudsen (2012) points out that a main strategy for managing the contingency of decisions in organizations is displacement. Communicating both inside and outside the organization to facilitate a development plan process was challenging for the managers. Various stakeholders, such as national, regional and local health authorities, as well as patients and healthcare professionals, activated different functional systems when seeking to understand the development plan, meaning that there was no shared understanding of what a development plan is or should be. To observe how organizations handle the paradox of decisions, the concepts inclusive management and brokers are used. The inclusive management literature emphasize on designing inclusive processes and creative a community of participation which also includes forums of sharing information among others (Feldman and Khademian, 2007). In line with Desmidt and Heene (2007), there was a gap between how the organization’s top management perceived the development plan and how the section managers and union representatives did. Top management was more comfortable working to meet political and economic goals, while these goals were too abstract and general for the professionals. Despite this gap, the managers assumed the role of brokers and tried to provide a shared experience and transcend the system boundaries between the participants so that the development plan statement process could bring about shared meaning. These strategies are in line with Carlie (2004) and Kimble *et al*. (2010).

However, the findings in this paper, shows that the managers did not succeed in their brokering role (Kimble *et al*., 2010). The problem was that the managers were not clear on what the decision premises for the development plan should be. In the plan process, several strategies were identified for managing the contingency of decisions. The managers tried to displace this contingency onto the political system by calling the development plan a political order, and they displaced this contingency onto the economic system by referring to the economic frames of the local health authorities. The emphasis on involving the healthcare professionals meant that this contingency was also situated inside the organization and was therefore not managed. As a result, some union representatives and section managers also tried to displace the contingency back onto the clinic management by urging them to clarify the decision premises for work on the development plan. When the decision premises were not managed, it was difficult to foster involvement, as it was not clear to the actors on what basis they should get involved. In line with Høiland and Klemsdal (2020) our case shows that conflicts in the plan process not only stems from the presences of multiple logics or codes, but also from differences within the organizations in how multiple logics are handled. Jansson *et al*. (2021) illustrate the same problems in decision-making processes during hospital hybridization. Decision makers tried to manage the paradox of decisions through different justification strategies, and by taking into account the different expectations of several societal systems, i.e. healthcare, education, science, law, economy and politics. Another reason involvement was difficult to achieve was the tension between control and autonomy (Lægerid *et al.*, 2008). On one side, the managers wanted to involve the whole organization, and the case shows many examples of both inclusive management and the managers taking the role of brokers. On the other side, the process of making a development plan is part of a larger political health project involving all levels of specialist healthcare. Connectivity is promoted throughout the multilevel plan process (Figure 2) by the health authorities different government levels. At the same time, the findings show that the local health service authorities emphasize that the local development plan must be based in the clinic perspective. Because the goal of involvement was set by the health authorities, it felt more like an obligation than the result of willing participation in a collaborative setting. Schwartz and Cohn (2002) shows that successful strategic planning can only occur with full participation. The clinic’s use of the “line principle” in involving the professionals as well as the various predefined guidelines enhanced the sense that the development plan work was a top-down process that emphasized control over autonomy.

## Conclusion

This study illustrates the theoretical point that it is impossible to get past the paradox of decisions. In our case, the managers’ different strategies for handling the contingency of decisions seemed to fail. One lesson from the study is, therefore, that managers, even before starting strategic planning processes, should reflect on how they intend to manage contingency and secure connectivity.

The results should also be seen in relation to the new public governance reforms that are influencing the health sector, in which integration, trust and equal collaboration is emphasized (Osborn, 2006). Managing complexity becomes a part of the interactive and collaborative nature of strategic planning plan making in today’s health sector. There are several positive aspects in this type of process where several stakeholder groups must cooperate. Through cross-professional exchange of experience, one can gain a new perspective and accumulate new knowledge that can stimulate a better process as well as end product. The processes themselves can also be an important part of an organization’s community. However, you cannot expect good processes and knowledge exchanges to happen automatically. The results of this study show the unreasonable demands placed on those who are to lead this type of planning process that requires both horizontal and vertical coordination. It especially pinpoints the challenges mid-level managers face. These managers must communicate the plan through different functional systems and to different stakeholders both inside and outside the organization. They also need to balance the governmental goal of control with the organization’s autonomy. At the same time there is an underlying expectation that multiple views should be anchored in the process and that the final product should be a result of something jointly produced. The study also illustrates managers' perceived difficulties related to fundamental factors such as time aspects, organization of work and knowledge acquisition. Many managers come from a health profession and are not formally trained in organizational development or coordination work. This together, places unreasonable demands on those who are to lead this type of planning process. Both practical training as well as more research is needed on how to deal with the complexity of coordinated plan practices in today’s health sector.

The findings from this study come from a case from Norway; however, many countries are undergoing reform changes in the public sector that are to be implemented on an organizational level. The strength of using case study method is that it may provide detailed, rich descriptions of complex problems that managers face in coordinated plan process. The case study methods also open for more theoretical explorations. However, it would be of interest to also see a study that takes a broader systematic approach, for example comparing similar processes in other clinics, organizations, sectors and/or countries. This to better get an idea of how these processes can be managed in a good matter.

This article has focused on how the development plans are managed at a clinical level. However, as shown in the findings, the plan work is set in a political context, in which regional and national government levels also play a role. We encourage more research on the multilevel governance plan process, and especially how plans might be adjusted from the clinic level back to the national government level. How is local knowledge taken account and what role does the local level have on regional and national government plans?

Making a development plan is often one step in structuring organizations that are undergoing changes. The conclusions presented in this article is of general interest and can be used in discussions with public sector managers working on strategy documents as well as policymakers to identify challenges that might hinder the implementation of political intentions.

## Figures and Tables

**Table 1:** Functional systems in society

System	Code	Medium	Program	Function
Political system	Government/opposition	Power	Ideology	Collective binding decisions
Economy	Payment/nonpayment	Money	Price	Distribution
Health	Ill/healthy	Illness	Diagnosis	Restoration
Science	True/untrue	Truth	Theory	Verification
Legal system	Lawful	Norms	Law	Standardization

**Source(s):** Adapted from Roth and Schutz (2015)
